# Valvuloplasty Without Prosthetic Ring or Band in Patients with Degenerative Mitral Regurgitation: Long-Term Results and Predictive Factors for Outcomes

**DOI:** 10.21470/1678-9741-2020-0520

**Published:** 2021

**Authors:** Renato A. K. Kalil, Karlyse C. Belli, Mariana O. T. de Mattos, Rita de Cássia E. Sffair, Sarah Ceolin Stein Santos, Vitória Recuero Fagundes, Rogério de Souza Abrahão, Álvaro Schmidt Albrecht, João Ricardo Michielin Sant'Anna, Paulo Roberto Prates, Ivo Abrahão Nesralla, Fernando Pivatto Júnior

**Affiliations:** 1 Cardiovascular Surgery Service, Instituto de Cardiologia do Rio Grande do Sul/Fundação Universitária de Cardiologia (IC/FUC), Porto Alegre, Rio Grande do Sul, Brazil.; 2 Surgery Department, Universidade Federal de Ciências da Saúde de Porto Alegre (UFCSPA), Porto Alegre, Rio Grande do Sul, Brazil.; 3 Internal Medicine Service, Hospital de Clínicas de Porto Alegre (HCPA), Porto Alegre, Rio Grande do Sul, Brazil.

**Keywords:** Mitral Valve/surgery, Mitral Valve Insufficiency, Mitral Valve Annuloplasty, Risk Factors, Treatment Outcomes

## Abstract

**Introduction:**

Mitral valvuloplasty including ring/band support is widely performed despite potential drawbacks of rings. Unsupported valvuloplasty is performed in only a few centers. This study aimed to report long-term outcomes of patients undergoing unsupported valvuloplasty for degenerative mitral regurgitation (MR) and to identify predictive factors for outcomes.

**Methods:**

This is a retrospective cohort including patients undergoing mitral valve repair for degenerative MR from 2000 to 2018. The main techniques were Wooler annuloplasty and quadrangular resection. Kaplan-Meier curves and Cox regression models were used for statistical analysis.

**Results:**

One hundred fifty-eight patients were included (median age: 64.0 years). In-hospital mortality was 2.5%. Maximum follow-up was 19.6 years, with a median of 4.7 years (992 patient-years). Overall survival at 5, 10, and 15 years was 91.0% (95% confidence interval [CI]: 85.7-96.3), 87.6% (95% CI: 80.7-94.5), and 78.1% (95% CI: 65.9-90.3), respectively. The European System for Cardiac Operative Risk Evaluation (EuroSCORE) II was an independent predictor of late death (hazard ratio [HR] 1.42; *P*=0.016). Freedom from mitral reoperation at 5, 10, and 15 years was 88.1% (95% CI: 82.0-94.2), 82.4% (95% CI: 74.6-90.2), and 75.7% (95% CI: 64.1-87.3), respectively. Left atrial diameter > 56 mm was associated with late reintervention in univariate analysis (HR 1.06; *P*=0.049).

**Conclusion:**

Degenerative MR can be successfully treated with repair techniques without annular support, thus avoiding the technical and logistical drawbacks of ring/band implantation while maintaining good long-term results. EuroSCORE II was a risk factor for late death, and larger left atrium was associated with late reoperation.

**Table:** 

Abbreviations, acronyms & symbols				
ACSD	= Adult Cardiac Surgery Database		LA	= Left atrial
			LV	= Left ventricular
ASD	= Atrial septal defect		LVEF	= Left ventricular ejection fraction
BMI	= Body mass index		MI	= Myocardial infarction
CABG	= Coronary artery bypass grafting		MR	= Mitral regurgitation
CCS	= Canadian Cardiovascular Society		NYHA	= New York Heart Association
CI	= Confidence interval		O/E	= Observed to expected
EuroSCORE	= European System for Cardiac Operative Risk Evaluation		SAM	= Systolic anterior motion
GFR	= Glomerular filtration rate		SD	= Standard deviation
HR	= Hazard ratio		sPAP	= Systolic pulmonary artery pressure
IQR	= Interquartile range		STS	= Society of Thoracic Surgeons

## INTRODUCTION

Mitral valvuloplasty is recognized as the treatment of choice for severe mitral regurgitation (MR) and indicated even in asymptomatic patients ^[^^1^^-^^3^^]^, with the possibility of restoring normal life expectancy ^[^^4^^]^. Most centers use rigid and flexible rings or posterior annular support bands in order to achieve stability, standardization, reproducibility, and durability of the results in valve repair ^[^^4^^,^^5^^]^.

Ring valvuloplasty, however, has drawbacks and potential risks, such as increased times of cardiopulmonary bypass and myocardial ischemia, as well as increased procedure costs. Implant size and implantation technique should be carefully chosen, as these features are associated with possible immediate and late complications, such as leaflet tethering, stenosis, poor adjustment to ring size, and subvalvular aortic stenosis due to poor ring sizing or systolic anterior motion (SAM) ^[^^6^^-^^8^^]^. Posterior bands and semicircular prosthetic rings minimize complications while maintaining the idea of support. However, although extensively reported in the literature and disseminated at events, none of these techniques is based on clinical evidence, such as randomized clinical trials.

Suture valvuloplasty without annular support has been performed systematically in only a few centers, but consistent results of this technique have been published for many decades ^[^^9^^-^^13^^]^. However, the dissemination of the results has been rather slow, possibly because of the great emphasis placed by centers of excellence on the need for prosthetic ring implantation, or even because of limited support received from interested entities, as there appears to be a shortage of sponsors for surgical techniques without the use of an implantable device.

The aim of this study was to present the long-term results regarding overall survival and freedom from mitral reoperation of patients with severe MR of degenerative etiology treated with valvuloplasty techniques without annular support of prosthetic rings or posterior bands.

## METHODS

We conducted a retrospective cohort study of consecutive patients undergoing mitral valvuloplasty without annular support for severe MR of degenerative etiology from 2000 to 2018. All procedures were performed by the same surgeon (R.A.K.K.). Patients were not included if they had MR of other etiologies, such as ischemic, functional, and rheumatic, or underwent surgery with implantation of rings or annular support bands. Patients with active endocarditis (still on antibiotic treatment at the time of surgery) ^[^^14^^]^ were excluded. First, eligible patients were identified by reviewing the operating room schedules. Subsequently, the patients’ medical records were reviewed to ensure that the study inclusion criteria were met. Follow-up data were collected from both electronic and paper medical records (outpatient visits, emergency department visits, and/or hospitalizations), as well as through direct contact with the patients’ attending physicians. The study was approved by the institution’s Research Ethics Committee.

The main surgical techniques used were quadrangular resection ^[^^15^^]^ with posterior annuloplasty and isolated leaflet monofilament sutures (Video 1), as needed, and Wooler annuloplasty ^[^^16^^]^ using sutures reinforced with Teflon felt anchored to the valve annulus at the two commissures (Video 2) in order to preserve the anterior leaflet width and reduce the posterior leaflet width, bringing the posterior leaflet toward the anterior leaflet while both undergo some degree of invagination toward the ventricle to achieve good coaptation. This bilateral technique of Wooler annuloplasty was used in cases of annular dilatation ^[^^16^^]^, *i.e.*, in cases without ruptured chordae tendineae or great prolapse of the posterior leaflet. The stitches were tied over pledgets from the fibrous trigones to the posterior direction, aiming to reduce the posterior annulus and achieve good coaptation. When necessary, additional isolated interventions were performed to correct specific defects in the leaflets or chordae, such as chordal shortening by direct suture plication. Also, in other cases, the indentations on the leaflet edges were joined with sutures in order to increase the anterior leaflet coaptation area. No posterior annular support band or any type of rigid or flexible ring was implanted in any of the patients.

**Video 1:**
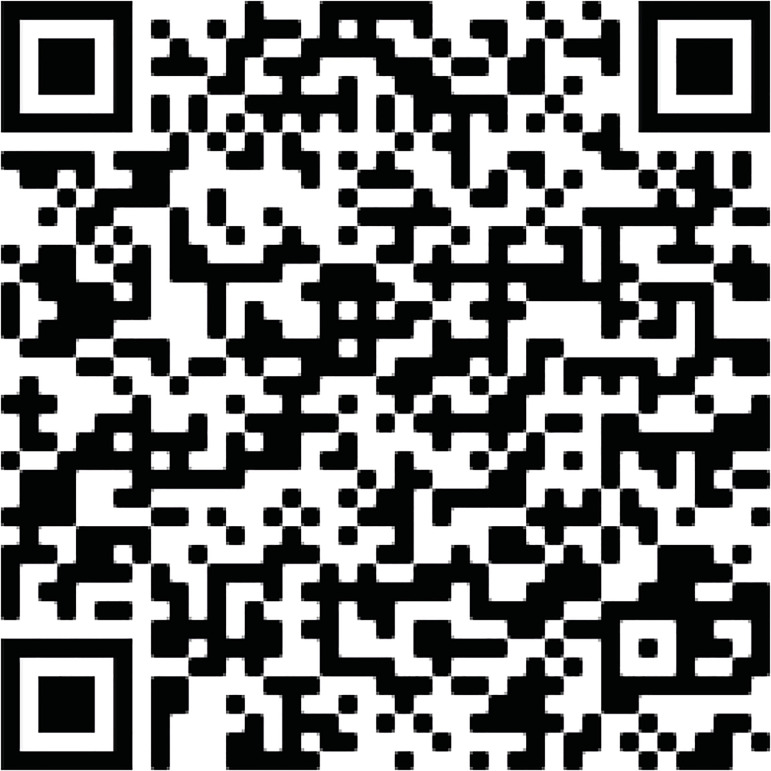
Quadrangular resection

**Video 2:**
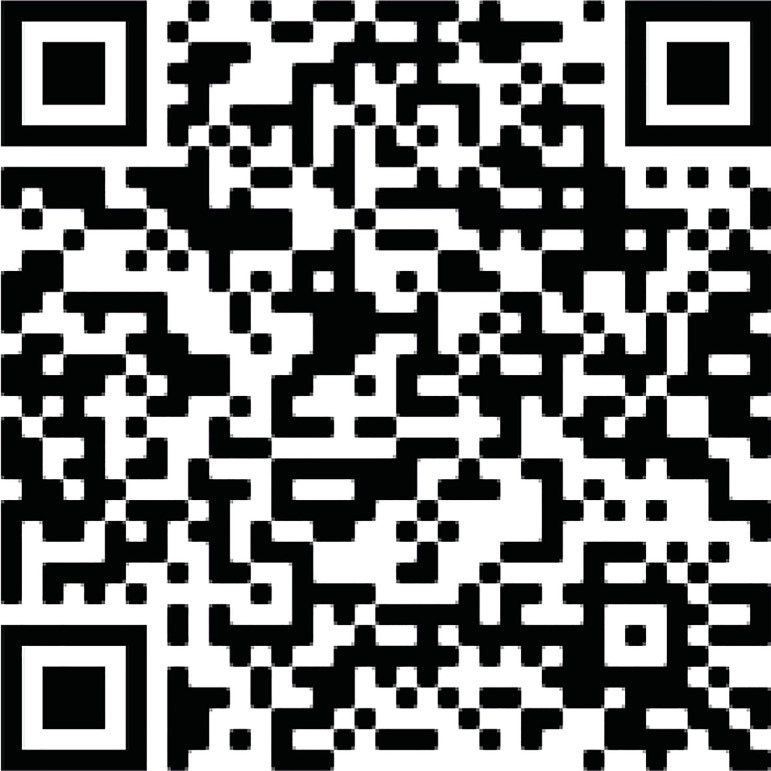
Wooler annuloplasty

It is important to note that the term “posterior annuloplasty”, as used herein, does not mean that a support band was added. Rather, it refers to situations where suture techniques are applied to the annulus relative to the posterior leaflet, as shown in the supplementary videos. When a quadrangular resection was made, the posterior annulus was restored with buttressed, double-armed sutures reinforced with large Teflon pledgets to reduce mitral circumference by reducing the posterior annulus width. However, when Wooler annuloplasty is mentioned, it means that the sutures are applied from the fibrous trigones to the posterior direction, thus reducing the posterior annulus width, advancing the posterior leaflet against the anterior leaflet to achieve close coaptation, and projecting both leaflets to the ventricular direction, which contributes to further increasing the coaptation surface.

The expected in-hospital mortality was calculated using the mean European System for Cardiac Operative Risk Evaluation (EuroSCORE) II score ^[^^14^^]^. The glomerular filtration rate was estimated using the Cockcroft-Gault formula ^[^^17^^]^. The critical preoperative state ^[^^14^^]^ was defined as the presence of any of the following conditions: acute renal failure (anuria or oliguria < 10 mL/h); cardiac massage; inotropic support; ventricular tachycardia or fibrillation or aborted sudden death; and mechanical ventilation or use of intra-aortic balloon counterpulsation/ventricular-assist device on arrival in the operating room. Extracardiac arteriopathy, chronic lung disease, pulmonary hypertension (systolic pulmonary artery pressure [sPAP] ≥ 30 mm Hg), recent myocardial infarction (< 90 days), poor mobility (severe impairment of mobility secondary to musculoskeletal or neurological dysfunction), and urgency of operation were defined according to EuroSCORE II ^[^^14^^]^.

### Statistical Analysis

Data were analyzed using MedCalc, version 12.5, OpenEpi, version 3.01 ^[^^18^^]^, and IBM Corp. Released 2012, IBM SPSS Statistics for Windows, Version 21.0, Armonk, NY: IBM Corp. For descriptive analysis, categorical variables were expressed as absolute and relative frequencies, continuous variables with normal distribution as mean ± standard deviation (SD), and continuous variables with non-normal distribution as median (interquartile range [IQR]). The Shapiro-Wilk test was used to test the normality of distribution. For between-group comparisons, categorical variables were compared by the chi-square test and quantitative variables were compared by unpaired Student’s *t*-test if normally distributed or by the Mann-Whitney U test if not normally distributed. Fisher’s exact test was used in case of low data frequency. The mid-P exact test with Miettinen’s modification was used to calculate the standardized mortality ratio, *i.e*., the ratio of observed to expected (O/E) mortality, with a 95% confidence interval (CI). Overall postoperative survival and survival free of mitral reoperation were analyzed using Kaplan-Meier curves compared across groups by the log-rank test. Cox regression models were used to identify predictors of survival, and variables with a *P*-value < 0.1 in the univariate analysis were included in the multivariate analysis. The best cutoff points for dichotomization of continuous variables were determined by the Youden index. The level of significance was set at 5%.

## RESULTS

A total of 158 patients were included, with a median age of 64 (IQR, 55.7-71.0) years; 61.4% were men. The baseline characteristics of the sample are shown in Table 1. No patient was in critical preoperative state. The median length of postoperative in-hospital stay was eight (IQR, 7-11) days.

**Table 1:** Patients' baseline characteristics.

Variable	n=158
Age (years)	64.0 (55.75-71.0)
Male	97 (61.4)
NYHA functional class III-IV	43 (27.2)
Atrial fibrillation	44 (27.8)
Previous cardiac surgery	10 (6.3)
Chronic lung disease	10 (6.3)
Diabetes on insulin	3 (1.9)
Recent MI (< 90 days)	2 (1.3)
CCS angina class 4	3 (1.9)
Extracardiac arteriopathy	4 (2.5)
Poor mobility	1 (0.6)
Cockcroft-Gault GFR (mL/min/1.73m^2^)	81.1 (61.4-107.7)
BMI (kg/m^2^)	24.6 (23.0-27.3)
EuroSCORE II (%)	1.3 (0.8-2.2)
**Echocardiographic data**	
Left atrium (mm)*	50.1±8.0
LV end-diastolic diameter (mm)^†^	60 (56-64)
LV end-systolic diameter (mm)^†^	37 (33-42)
LVEF (%)	67 (61-73)
sPAP ≥ 30 mmHg	68 (43.0)

BMI=body mass index; CCS=Canadian Cardiovascular Society; EuroSCORE=European System for Cardiac Operative Risk Evaluation; GFR=glomerular filtration rate; LV=left ventricular; LVEF=left ventricular ejection fraction; MI=myocardial infarction; NYHA=New York Heart Association; sPAP=systolic pulmonary artery pressure

* 15 (9.5%) and

† 16 (10.1%) patients without available data Data expressed as n (%), mean & #x00b1; standard deviation, or median (interquartile range)

Most patients (n=152, 96.2%) underwent elective mitral repair. Associated surgery occurred in 56 (35.4%) patients, being coronary artery bypass grafting the most frequent procedure (n=24, 42.9%). Surgical data are shown in Table 2.

**Table 2:** Surgical data.

Variable	n=158
Elective surgery	152 (96.2)
Cross-clamping time (min)	36 (29-45)
Cardiopulmonary bypass time (min)	51 (42-65)
Associated surgery*	56 (35.4)
CABG	24 (15.2)
Pulmonary vein isolation	21 (13.3)
ASD repair	5 (3.2)
LA appendage excision	5 (3.2)
Tricuspid valve repair	3 (1.9)
Mechanical aortic valve replacement	3 (1.9)
LV aneurysmectomy	1 (0.6)
Aortoplasty (ascending aortic aneurysm)	1 (0.6)
LA appendage closure	1 (0.6)
Aortic valve repair	1 (0.6)
Biological aortic valve replacement	1 (0.6)
Subaortic resection	1 (0.6)

ASD=atrial septal defect; CABG=coronary artery bypass grafting; LA=left atrial; LV=left ventricular

* ≥ 1 associated procedure Data expressed as n (%) or median (interquartile range)

In-hospital mortality was 2.5% (95% CI: 0.1-4.9). This rate was 2.0% in patients undergoing isolated mitral valvuloplasty and 3.6% in patients undergoing concomitant procedures (*P*=0.615). The expected mortality calculated by EuroSCORE II was 1.8%, with an O/E mortality ratio of 1.4 (95% CI: 0.4-3.4; *P*=0.46). Cardiogenic, septic, or mixed (cardiogenic and septic) shock and extensive stroke were the causes of death.

**Fig. 1:**
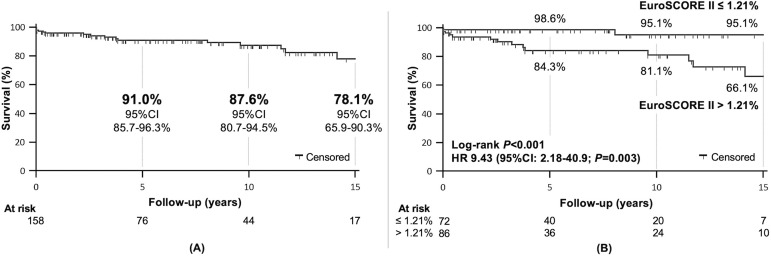
(A) Overall postoperative survival. (B) According to the European System for Cardiac Operative Risk Evaluation (EuroSCORE) II. CI=confidence interval; HR=hazard ratio

The median postoperative follow-up duration was 4.7 (IQR, 1.7-10.5) years (992 patient-years), with a maximum follow-up of 19.6 years. Eighteen (11.7%) patients died after hospital discharge, seven (38.9%) of cardiovascular causes. The actuarial postoperative survival curve at 5, 10, and 15 years is shown in Figure 1A. Of all variables analyzed, only EuroSCORE II was an independent predictor of mortality (Table 3). EuroSCORE II > 1.21% was the best cutoff point, associated with higher mortality (Figure 1B) during follow-up (hazard ratio [HR] 9.43; *P*=0.003).

**Table 3:** Univariate and multivariate analyses.

Variable	Univariate analysis		Multivariate analysis	
	HR (95% CI)	*P*-value	HR (95% CI)	*P*-value
Overall postoperative survival				
EuroSCORE II (%)	1.38 (1.12-1.70)	.002	1.42 (1.07-1.90)	0.016
CCS angina class 4*	6.00 (1.33-27.0)	.02	-	-
Associated surgery*	3.19 (1.30-7.80)	.011	-	-
Age (years)*	1.06 (1.01-1.11)	.023	-	-
LV end-diastolic diameter (mm)	1.02 (1.00-1.05)	.047	1.01 (0.99-1.03)	0.355
Cardiopulmonary bypass time (min)	1.01 (0.99-1.03)	.066	1.01 (0.99-1.03)	0.374
Cross-clamping time (min)^†^	1.02 (1.001-1.05)	.039	-	-
Overall postoperative survival free of mitral reoperation				
EuroSCORE II (%)	1.28 (0.997-1.63)	.053	0.91 (0.48; 1.71)	0.763
CCS angina class 4*	12.2 (2.71-55.2)	.001	-	-
Associated surgery*	3.0 (1.23-7.37)	.016	-	-
NYHA functional class III-IV*	2.81 (0.91-8.67)	.072	-	-
Left atrium (mm)	1.06 (1.00-1.12)	.049	1.01 (0.93-1.10)	0.810
sPAP (mm Hg)	1.04 (0.999-1.08)	.058	1.05 (0.998-1.11)	0.057
Cardiopulmonary bypass time (min)	1.02 (1.00-1.03)	.051	1.00 (0.966-1.03)	0.910
Cross-clamping time (min)^†^	1.02 (0.99-1.04)	.067	-	-

CCS=Canadian Cardiovascular Society; CI=confidence interval; EuroSCORE = European System for Cardiac Operative Risk Evaluation; HR=hazard ratio; LV=left ventricular; NYHA=New York Heart Association; sPAP=systolic pulmonary artery pressure

* Variable not included in the multivariate analysis as it is already part of EuroSCORE II

† Because there is multicollinearity with cardiopulmonary bypass time, we decided not to include cross-clamping time in the multivariate analysis

During postoperative follow-up, there were 20 (12.7%) cardiac reoperations involving the mitral valve. Isolated biological valve replacement (n=11, 55.0%) was the main procedure performed, and severe valvular regurgitation (n=11, 55.0%) was the main indication for reintervention (Table 4).

**Table 4:** Mitral reoperations during follow-up and indications.

Variable	n=158
**Mitral reoperations**	**20 (12.7)**
Isolated biological mitral prosthesis	11 (7.0)
Isolated mechanical mitral prosthesis	4 (2.5)
Isolated mitral repair	1 (0.6)
Mitral repair + CABG	1 (0.6)
Mitral repair + biological tricuspid prosthesis	1 (0.6)
Mitral repair + mechanical aortic prosthesis	1 (0.6)
Mitral repair + lung cancer resection	1 (0.6)
**Reoperation indications**	
Severe MR	11 (55.0)
Moderate MR	5 (25.0)
Severe combined MR and stenosis	4 (20.0)

CABG=coronary artery bypass grafting; MR=mitral regurgitation Data expressed as n (%) or median (interquartile range).

**Fig. 2:**
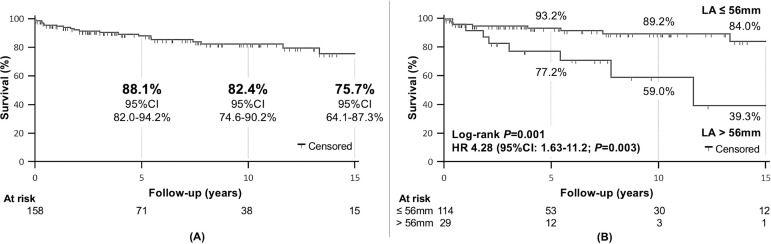
(A) Overall postoperative survival free of mitral reoperation. (B) According to left atrial (LA) diameter. CI=confidence interval; HR=hazard ratio

The curve for postoperative survival free of mitral reoperation is shown in Figure 2A. Of all preoperative echocardiographic data analyzed (Table 1), only left atrial (LA) diameter was associated with mitral reoperation during follow-up in univariate analysis (Figure 2B). LA diameter > 56 mm was the best cutoff point, associated with a significantly higher reoperation rate (HR 4.28; *P*=0.003). However, no independent predictor of reoperation was identified in multivariate analysis (Table 3).

## DISCUSSION

The present study included all patients operated on consecutively for MR of degenerative etiology over a period of 19 years using reconstructive techniques without ring implantation or annular support bands, as used by the authors since 1973. In previous publications, we have reported our experience with these techniques in MR of rheumatic ^[^^11^^]^ and congenital etiologies ^[^^19^^]^, as well as our experience in the first three decades of the degenerative etiology ^[^^20^^]^. Since then, there have been changes in the prevalence of MR etiologies in the reference region of the hospital under study, with a significant reduction in the rheumatic etiology and current predominance of the degenerative etiology. Without underestimating the preference of most centers dedicated to mitral valvuloplasty for prosthetic ring implantation, which is considered by some to be essential, our experience gained over the years of practice in the specialty, associated with the growing emphasis in recent decades on prioritizing mitral valve repair over replacement, prompted us to review the results of mitral valve repair procedures performed in a center that does not prioritize ring implantation; on the contrary, it does not even use ring implantation as part of the routine valve reconstruction procedures for MR.

In degenerative MR, the mitral annulus is consistently reported as enlarged, flattened, and more circular, with increased anteroposterior diameter, intercommissural diameter, circumference, and area compared to normal valves, whereas annulus height is close to normal ^[^^21^^]^. The abovementioned morphopathological characteristics of MR are well recognized, but they do not significantly influence the choice of surgical technique. In the presence of increased intercommissural distance relative to the anterior annulus, between both fibrous trigones, that distance is maintained in order to preserve the entire anterior leaflet width. Preserving the entire anterior leaflet is essential for the success of unsupported valvuloplasty. When reducing the posterior annulus by suturing close to the commissures or after quadrangular resection, both valve circumference and area are reduced; this is done until proper leaflet apposition with an acceptable coaptation area is achieved.

The findings of overall survival of 87.6% at 10 years and 78.1% at 15 years, associated with survival free of mitral reoperation of 82.4% at 10 years and 75.7% at 15 years, attest to the stability and durability of the procedures and are comparable to those of most published case series prioritizing ring implantation. As a secondary finding, EuroSCORE II was not only a predictor of in-hospital morbidity and mortality but was also significantly associated with long-term overall survival, which provides important information for clinical guidance. In addition, we believe that the association between LA diameter and the likelihood of late reoperation is an important finding, although it was significant only in univariate analysis. The pathophysiological mechanism responsible for such behavior is not fully understood and warrants further investigation. The literature reviewed for this study does not mention the two factors abovementioned as predictors of outcomes. The well-described factors reported to influence the long-term durability of degenerative MR repair include advancing age, left ventricular dysfunction (ejection fraction < 60% or end-systolic dimension > 40 mm), New York Heart Association III-IV, and permanent atrial fibrillation ^[^^22^^]^.

One issue that can be brought up for discussion is the center’s surgical volume and its relationship with the results of mitral valvuloplasty. The present series consists of 158 patients operated on over a 19-year period, that is, an average of 8.3 cases per year. However, this number does not account for all cases of mitral valve surgery performed in the period, as cases of rheumatic, congenital, and functional MR were not included, nor were the cases of valve replacement, infectious endocarditis, and other conditions that were not exclusively related to the repair of degenerative MR. According to data from the Society of Thoracic Surgeons (STS) Adult Cardiac Surgery Database (ACSD) ^[^^23^^]^, published in 2018, of the 1,143 participating centers in the United States of America, only 256 (22.4%) treated more than six cases of degenerative MR per year, and only 15 centers in the country treated more than 50 cases per year. Therefore, the volume of mitral operations performed at our center is comparable to or higher than the 77.6% of the participating centers in the STS ACSD.

It is important that the reasons for our preference for avoiding ring implantation are discussed, as this approach contradicts the prevailing consensus in international practice. Initially, it should be noted that our experience precedes or is at least coincident with the advent of the proposed use of a prosthetic ring to support the valve annulus, much emphasized by the school competently led by Carpentier ^[^^5^^,^^24^^]^, whose work must be recognized as fundamental in knowledge dissemination and in the training of surgeons in valvuloplasty techniques. Valve repair in MR inevitably aroused some suspicion as to its reproducibility and durability, and it was only in recent decades that it was recognized and prioritized as the technique of choice, to the point of being indicated even in asymptomatic patients in current guidelines. In a parallel experience, our center and others ^[^^9^^,^^10^^,^^12^^,^^13^^,^^20^^]^ chose not to adhere to the “mandatory” prosthetic ring implantation. The results obtained at that time were comparable to those published by authors who preferred ring implantation, and they continue to be today, as shown in this report.

In addition to the very long-term results with ring-based techniques previously mentioned ^[^^5^^]^, some recent reports also deserve mention. Mohty et al. ^[^^25^^]^ followed a cohort of 917 patients with severe MR operated on between 1980 and 1995 (mean follow-up of 7.7±4.1 years). Mitral valve repair was performed in 679 patients (74.0%) and involved subvalvular (chordal shortening or artificial chord insertion), valvular (mostly resection or plication), and annular (mostly ring insertion) interventions. Overall survival rates after mitral valve repair were 86% (95% CI: 84-88) at five years, 68% (95% CI: 64-72) at 10 years, and 37% (95% CI: 27-47%) at 15 years, whereas the need for reoperation was 93% (95% CI: 91-95), 89% (95% CI: 85-93), and 84% (95% CI: 78-90), respectively. In a randomized controlled trial, Chang et al. ^[^^26^^]^ evaluated 356 patients surgically treated for MR (degenerative etiology: 236 [66.3%]) and described a reoperation-free survival of 99.3% (95% CI: 96.0-100) at five years and 77.4% (95% CI: 61.7-93.1) at 10 years. David et al. ^[^^27^^]^ reported the results of the follow-up of 701 patients with degenerative MR who underwent valvuloplasty between 1981 and 2001; rings or support bands were used in 668 (95.3%) cases. Mean (±SD) follow-up was 6.9±4.0 years. Overall survival at 12 years was 75% (95% CI: 65-85) and survival free of severe recurrent MR was 89% (95% CI: 85-93); these data can be compared to the reoperation-free survival reported in the present series, since in this study all cases of severe recurrent MR were reoperated.

The drawbacks of ring implantation can be minimized by implanting posterior annular support bands in the space between the two fibrous trigones. This has been the practice of many authors, with the use of prefabricated semicircular prosthetic rings ^[^^28^^,^^29^^]^ or simple bands made of Dacron, bovine pericardium, or autologous pericardium. In the view and experience of this center, however, even band implantation is not necessary to maintain the stability of the repair, although it can bring more emotional support to the surgeon.

It is outside the scope of this report to give a detailed account of the various models of implanted rings, whether rigid or flexible, complete or partial. All devices are well known and have the proposed advantage of providing repair stability. However, disadvantages are also apparent and include longer operative and myocardial ischemia times due to the increased complexity of the technique. There is a non-negligible increase in costs, which, although secondary when the goal is a good clinical outcome, should always be considered. Choosing the proper ring type and size requires technique and art ^[^^6^^,^^7^^]^, as, if not properly done, it can result in immediate and late complications ^[^^8^^,^^29^^]^. The rings fix the mitral annulus and prevent its contraction. Three-dimensional analysis shows that the mitral valve plane is saddle shaped ^[^^30^^]^ and has a dynamic behavior. At the end of the ventricular systole, the surface of the mitral valve orifice is reduced by up to 30% of its area, a phenomenon that contributes to valve competence. If a prosthetic ring is placed, both the shape and dynamics can be altered, thus changing the physiology of the mitral valve apparatus. Altered dynamics can lead to the frequent phenomenon of SAM of the anterior mitral leaflet, with consequent dynamic subvalvular aortic stenosis. On the other hand, placing an oversized ring may also result in subvalvular aortic stenosis. Finally, other reported disadvantages of prosthetic ring implantation include late progressive mitral stenosis ^[^^31^^,^^32^^]^ and other dysfunctions capable of causing valvular regurgitation ^[^^6^^]^.

### Limitations

Limitations of this study include the relatively small sample size and those inherent in its retrospective design, which can affect the quality of the analyzed data. In addition, events (death/mitral reoperation) may have been underreported due to losses to follow-up. Finally, the fact that this is a single-center study ensures uniformity in the follow-up of the patients included in this cohort, but it can reduce the external validity of the findings.

## CONCLUSION

In conclusion, the data described in this series of consecutive cases of valve repair for MR of degenerative etiology allow us to conclude that it is possible to perform mitral valve repair in a safe and stable manner without the implantation of posterior support bands or even rings, whether rigid or flexible, complete or partial. Actuarial overall survival and survival free of mitral reoperation were satisfactory and comparable to those of series describing the use of annular support. The main advantages of unsupported valvuloplasty include the maintenance of the three-dimensional shape and physiology of the mitral valve orifice, accompanying systole and diastole in the normal cardiac cycle, without causing fixed or dynamic stenosis in the left ventricular outflow tract. In addition to the factors known to influence repair durability already described in the literature, we found high EuroSCORE II and LA diameter to be risk factors for mortality and late reoperation, respectively. Unsupported mitral valve repair also offers the advantage of shorter operative and aortic cross-clamping times, in addition to lower hospital costs.

**Table:** 

Authors' roles & responsibilities	
RAKK	Substantial contributions to the conception or design of the work; or the acquisition, analysis, or interpretation of data for the work; agreement to be accountable for all aspects of the work in ensuring that questions related to the accuracy or integrity of any part of the work are appropriately investigated and resolved; drafting the work or revising it critically for important intellectual content; final approval of the version to be published
KCB	Substantial contributions to the conception or design of the work; or the acquisition, analysis, or interpretation of data for the work; final approval of the version to be published
MOTM	Substantial contributions to the conception or design of the work; or the acquisition, analysis, or interpretation of data for the work; final approval of the version to be published
RCES	Substantial contributions to the conception or design of the work; or the acquisition, analysis, or interpretation of data for the work; final approval of the version to be published
SCSS	Substantial contributions to the conception or design of the work; or the acquisition, analysis, or interpretation of data for the work; final approval of the version to be published
VRF	Substantial contributions to the conception or design of the work; or the acquisition, analysis, or interpretation of data for the work; final approval of the version to be published
RSA	Final approval of the version to be published
ASA	Final approval of the version to be published
JRMS	Final approval of the version to be published
PRP	Final approval of the version to be published
IAN	Final approval of the version to be published
FPJ	Substantial contributions to the conception or design of the work; or the acquisition, analysis, or interpretation of data for the work; agreement to be accountable for all aspects of the work in ensuring that questions related to the accuracy or integrity of any part of the work are appropriately investigated and resolved; drafting the work or revising it critically for important intellectual content; final approval of the version to be published
